# Influence of mental health and alcohol or other drug use risk on adolescent reported care received in primary care settings

**DOI:** 10.1186/s12875-017-0689-y

**Published:** 2018-01-09

**Authors:** Lisa S. Meredith, Brett A. Ewing, Bradley D. Stein, William G. Shadel, Stephanie Brooks Holliday, Layla Parast, Elizabeth J. D’Amico

**Affiliations:** 1 RAND Corporation, 1776 Main Street, Santa Monica, CA 90407-2138 USA; 2 VA HSR&D Center for the Study of Healthcare Innovation, Implementation, and Policy, California, Los Angeles USA; 3 RAND Corporation, Pittsburgh, PA USA

**Keywords:** Screening and brief intervention, Adolescents, Primary care

## Abstract

**Background:**

To describe patterns of alcohol and other drug (AOD) use risk and adolescent reported primary care (PC) screening and intervention, and examine associations of AOD risk and mental health with reported care received.

**Methods:**

We analyzed data from cross-sectional surveys collected from April 3, 2013 to November 24, 2015 from 1279 diverse adolescents ages 12–18 who reported visiting a doctor at least once in the past year. Key measures were AOD risk using the Personal Experience Screening Questionnaire; mental health using the 5-item Mental Health Inventory; binary measures of adolescent-reported screening and intervention.

**Results:**

Half (49.2%) of the adolescents reported past year AOD use. Of the 769 (60.1%) of adolescents that reported being asked by a medical provider in PC about AOD use, only 37.2% reported receiving screening/intervention. The odds of reported screening/intervention were significantly higher for adolescents with higher AOD risk and lower mental health scores.

**Conclusions:**

Adolescents at risk for AOD use and poor mental health are most likely to benefit from brief intervention. These findings suggest that strategies are needed to facilitate medical providers identification of need for counseling of both AOD and mental health care for at risk youth.

**Trials registration:**

clinicaltrials.gov, Identifier: NCT01797835, March 2013.

## Background

Rates of alcohol and other drug use (AOD) continue to be high [1] and adolescent AOD use is associated with significant health and social consequences [2–8]. AOD use also increases risk of having an AOD disorder as an adult [9], and is associated with high economic and societal costs [10, 11]. Because most adolescents seek care in a primary care (PC) setting at least once a year [12–14], early identification or treatment by PC clinic staff is a promising way to prevent or ameliorate AOD among adolescents [15], and multiple guidelines recommend such screening [16–18]. However, rates of screening and intervention for AOD in PC are low for adolescents [19–22]. Some of the reasons for low AOD screening rates are that providers are unsure of the importance of prevention and approval of alcohol screening [23], limited organizational support, lack of training, discomfort [24], and limited opportunity [25]. Other obstacles include lower provider perceived alcohol-management skills, and lower provider self-efficacy [23, 25].

National surveys on adolescent AOD use and mental health problems consistently find overlap between these disorders [26–28]. Adolescent reports of AOD use and mental health symptoms also co-occur [29]. To our knowledge, the potential additive or interactive effect of having poor mental health and risky AOD use on PC screening/intervention has not been examined. We address this gap in the literature, by examining the association between AOD use and adolescent-reported screening/intervention by medical providers in PC settings. Our research questions were: 1) Are adolescents with greater risk for AOD more likely to report being screened by a medical provider in PC for AOD use and followed up with intervention compared to adolescents with less risk for AOD use? 2) Does having poorer mental health in addition to higher AOD risk increase the odds that adolescents will report receiving an intervention in a PC setting to address AOD risk?

## Methods

### Study settings

The study sample was recruited from PC clinics providing care predominantly for ethnically and racially diverse and underserved adolescents in Pittsburgh, PA (3 clinics) and Los Angeles, CA (1 clinic). These sites offer longitudinal, continuity-based care and episode-based urgent care to their patients. Clinics in both areas have a large percentage of minority patients and low-income patients who are uninsured or underinsured (i.e., insured through Medicare and/or Medicaid).

### Participants and procedures

Every adolescent between age 12 and 18 in the clinic waiting rooms with a scheduled appointment was asked to be part of the project. For interested adolescents, we obtained parental consent and assent (if under 18) or consent if 18. Youth were screened by project staff using the National Institute of Alcohol and Alcohol Abuse (NIAAA) screening guide, completed a survey via the web from April 2013 to November 2015, and were paid $25. We obtained a certificate of confidentiality; the Institutional Review Boards and the clinics approved procedures.

Approximately 3309 adolescents were approached to participate; 27% (*n* = 892) were ineligible because they were not 12–18 years old, not proficient in English, were not at the clinic for their own appointment, or were disabled, and 18.5% (*n* = 614) refused to participate (mostly because of adolescents’ concerns that their parents would learn they were at the clinic for a family planning appointment or because of time constraints). After exclusions, 1803 adolescents enrolled or consented for study staff to contact them. Of the 1803 adolescents, 230 did not complete the baseline survey within the field period or had inaccurate contact information. This yielded a final sample of 1573 adolescents. The average age was 15.5; 42.5% were male; 51.4% were Hispanic, 26.7% black, 14.8% white, and 7.2% multiethnic or other. We focus on 1279 adolescents who reported visiting the doctor at least once in the past year and also had usable information to allow categorization into an AOD risk category.

### Measures

We used items from a longitudinal national household survey to assess five main outcomes of self-reports of screening and intervention [30, 31]. Adolescents were asked, “In the past 12 months, did any medical provider (e.g., primary care physician, general internist, nurse, physician assistant, chiropractor, or health clinic): 1) ask about use of alcohol or drugs (screening), 2) suggest cutting down or stopping use of alcohol or drugs (education/advice intervention), 3) suggest seeing a specialist or special program for emotions, mental health, or alcohol or drug use (referral intervention), or 4) provide counseling about emotions, mental health, alcohol or drug use for at least 5 minutes (counseling intervention).” We created binary indicators for each of these outcomes plus a fifth indicator for receipt of screening plus at least one type of intervention.

We assessed AOD risk using the 18-item Problem Severity Scale of the Personal Experience Screening Questionnaire (PESQ-PS) [32] that is reliable and valid for use in a general adolescent population. The PESQ-PS consists of eight items about AOD use in different contexts, nine items about behaviors and consequences of AOD use, and a single item about selling drugs to pay for AOD use. Items were rated on a 4-point frequency scale (never, once or twice, sometimes, or often). We used validated cut-points on the summed score (ranging from 18 to 72; α = 0.93) to stratify adolescents into risk groups. Low risk or green flag suggests no further assessment or referral is recommended because the individual has no problem with AOD use. Medium risk or yellow flag suggests that the individual has a mild or moderate problem with AOD use requiring brief intervention. High risk or red flag suggests abusive or dependent use of AOD requiring comprehensive evaluation to confirm severity of the problem with likely referral for substance abuse treatment. Cut-points by gender and age range were >23–24 for yellow flag and >30–35 for red flag.

We measured mental health using the well-validated 5-item Mental Health Inventory (MHI-5) [33]. Respondents indicated frequency for five feelings during the past month on a 1–6 scale (all of the time to none of the time). Item content included feeling nervous, calm and peaceful, downhearted and blue, happy, and down in the dumps. Relevant items were reversed so that a higher total score (transformed to range from 0 to 100; α = 0.73) indicated better mental health.

We controlled for adolescent age, gender, indicators of race/ethnicity, and mother’s education (less than college vs. at least some college)**.** We imputed incomplete data for mother’s education using an ordinal logistic regression model with race/ethnicity, city, and living in a two-parent household [34, 35].

### Analysis

We first describe sample characteristics, patterns of, and rates of screening and intervention overall and by risk AOD groups by the PESQ-PS. We use multivariable logistic regression to estimate odds of receiving screening and each type of intervention as a function of AOD use risk and mental health, controlling for demographic characteristics (age, gender, race, and mother’s education). To understand the independent effect of AOD risk, the effect of AOD risk after controlling for mental health, and the interactive effect of AOD risk and mental health, we ran three sets of models: (1) including only the main effect for AOD risk groups without the MHI-5, (2) adding the main effect for MHI-5, and (3) adding the interaction between AOD risk and MHI-5. We conducted analyses using standard statistical software (SAS 9.3). We adjusted for clustering of adolescents within clinics using fixed effects for clinics.

## Results

### Sample characteristics

This analysis sample (*n* = 1279) was similar to the full enrolled sample (*n* = 1573) (Table 1): average age of 15.6 years, 40.4% male, 26.3% Black, 52.0% Hispanic, and 6.6% multiethnic. Most mothers (67.1%) had no college education. The average score on the MHI-5 was 68.5. All characteristics differed significantly across PESQ-PS risk groups except for mothers’ education. Adolescents at highest risk (red flag) were significantly older (*p* < .01), more likely to be female (*p* < .001), more likely to be Hispanic (p < .001), and reported lower mental health scores (p < .001).

**Table 1:** Adolescent Sample Characteristics by AOD Risk Groups on the PESQ

	Full Sample (n = 1279)	PESQ Risk Group		
		Green Flag (*n* = 886)	Yellow Flag (*n* = 195)	Red Flag (*n* = 221)
Age**	15.6 (0.2)	15.2 (0.2)	16.3 (0.1)	16.7 (0.1)
Gender***				
Male	40.4	42.1	40.9	33.2
Female	59.6	57.9	59.1	66.8
Race***				
White	15.0	14.8	16.6	14.5
Black	26.3	28.5	24.9	19.1
Hispanic	52.0	48.8	55.4	61.4
Multiethnic/Other	6.6	7.9	3.1	5.0
Mother’s Education				
Less than college	67.1	65.2	67.9	73.4
At least some college	32.9	34.8	32.1	26.6
MHI-5 Score (0–100)***	68.5 (1.0)	69.9 (0.7)	70.3 (2.0)	61.5 (1.0)

*AOD* Alcohol or drug use, *PESQ* Personal Experience Screening Questionnaire; 228 cases were imputed for missing data on mother’s education; Analyses are adjusted for clustering within clinic; **p < .01; ***p < .001

### Patterns of screening and intervention

**Fig. 1:**
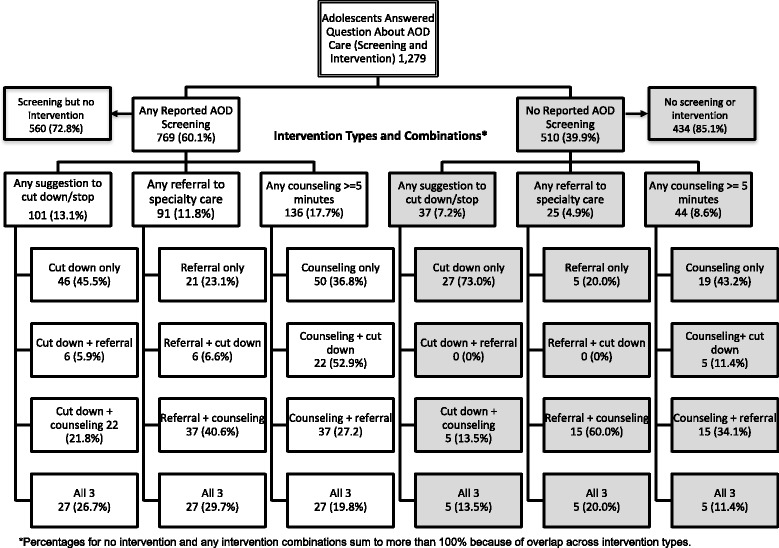
Patterns of Past Year Adolescent Reported AOD Care (Screening and Intervention) Received by Medical Providers

Figure 1 illustrates the flow of screening and interventions as reported by the 1279 adolescents who answered the question about care received (screening and intervention) in a past year doctor visit. Of the 769 adolescents who reported being asked about AOD use (e.g., any reported AOD screening), 72.8% (*n* = 560) reported being screened for AOD use by the doctor but received no further intervention, and 209 reported being screened and at least one type of intervention. Of those 769 adolescents, only 13.1% (*n* = 101) reported being asked to cut down or stop using AOD, 11.8% (*n* = 91) reported being referred to a mental health specialist or substance use program, and 17.7% (*n* = 136) reported receiving counseling for at least five minutes about mental health or AOD. Limited numbers of adolescents reported receiving multiple interventions, with only 27 receiving all three interventions. Almost 40% of adolescents reported that they were not screened about AOD use, and the 510 adolescents, the majority received no intervention 85.1% (*n* = 434). But, 7.2% (*n* = 37) of unscreened adolescents reported being asked to cut down or stop using AOD, 4.9% (*n* = 25) reported being referred to a mental health specialist or substance use program, and 8.6% (*n* = 44) reported receiving counseling for at least five minutes about mental health or AOD.

### Rates of screening and intervention

**Fig. 2:**
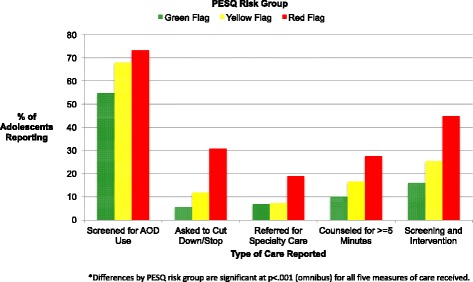
Adolescent Reported Care Received by AOD Risk Group*

Rates of screening and intervention as reported by adolescents were significantly higher with increased risk (omnibus test, *p* < .001 for all) (Fig. 2). Adolescents in the red flag group were 18% more likely to be screened for AOD use compared with the green flag group (*p* < 0.001). For intervention, 25% more red flag adolescents reported being advised to cut down or stop use than were green flag adolescents (*p* = 0.003). This difference was 12% more for referral (*p* = 0.016) and 18% more for counseling (*p* = 0.002). Red flag adolescents were also 29% more likely to report screening plus intervention relative to green flag adolescents (p = 0.002).

### Associations between AOD risk and mental health with screening and intervention

Table 2 shows the results for multivariable logistic regression models estimating the likelihood of adolescent reported screening and intervention received from medical providers after adjusting for clustering within clinic and demographic characteristics. Models include the main effects for both MHI-5 and AOD risk (without the interaction). Better mental health was not significantly associated with adolescents reporting being asked about AOD use or advice to cut down or stop using AOD, but was significantly associated with a slightly lower likelihood of reporting being referred, counseled, or getting screened and receiving any type of intervention (all OR = 0.97–0.98, *p* < .001). Higher AOD risk was significantly associated with increased likelihood of receiving screening and all types of intervention for red flag vs. green flag (all p < .001) and with suggesting cutting down (*p* < .01), counseling (p < .001), and screening plus any intervention (p < .001) for yellow flag vs. green flag. Compared to the green flag group, adolescents in the red flag group were 1.7 times more likely to report being screened, 8.9 times more likely to report being advised to cut down or stop AOD use, 2.8 times more likely to be referred, 3.5 times more likely to be counseled for at least five minutes, and 4.4 times more likely to have been both screened and received an intervention.

**Table 2:** Odds Ratios for Adolescent Reported Screening or Intervention Received by Medical Providers in Primary Care

	Screening or Intervention (Dependent Variable)				
	Asked about AOD use	Suggested cutting down or stopping AOD use	Referred for specialty care	Counseled for > = 5 min	Asked and provided any treatment
Independent Variable	OR [95% CI]	OR [95% CI]	OR [95% CI]	OR [95% CI]	OR [95% CI]
MHI-5 Score (0–100)	1.01 [1.00–1.01]	1.01 [1.00–1.02]	0.97 [0.96–0.98]***	0.97 [0.97–0.98]***	0.98 [0.97–0.99]***
PESQ Risk Flags					
Red (vs. Green)	1.69 [1.19–2.40]**	8.89 [5.59–14.14]***	2.80 [1.73–4.54]***	3.48 [2.29–5.30]***	4.44 [3.11–6.36]***
Yellow (vs. Green)	1.36 [0.96–1.92]	2.37 [1.37–4.09]**	1.14 [0.61–2.14]	2.15 [1.35–3.42]***	2.03 [1.37–3.01]***
Age	1.25 [1.17–1.34]***	0.98 [0.88–1.11]	0.96 [0.85–1.08]	0.88 [0.80–0.97]*	0.93 [0.85–1.01]
Gender					
Female (vs. Male)	1.19 [0.93–1.52]	0.61 [0.41–0.91]*	0.81 [0.53–1.25]	0.93 [0.65–1.33]	0.81 [0.60–1.08]
Race					
Black (vs. White)	0.42 [0.27–0.66]***	1.03 [0.51–2.08]	1.00 [0.50–1.99]	0.91 [0.50–1.68]	0.80 [0.48–1.33]
Hispanic (vs. White)	0.40 [0.26–0.64]***	0.98 [0.49–1.97]	1.17 [0.58–2.33]	1.12 [0.62–2.01]	0.93 [0.57–1.54]
Multi/Other (vs. White)	0.56 [0.31–0.99]*	1.05 [0.41–2.69]	0.88 [0.32–2.38]	1.46 [0.69–3.10]	1.12 [0.58–2.13]
Mother’s Education					
At least some college (vs. less than college)	1.07 [0.81–1.41]	0.93 [0.59–1.45]	1.10 [0.69–1.76]	1.31 [0.89–1.93]	1.22 [0.88–1.69]
Clinic					
Clinic A (vs. Clinic D)	0.80 [0.52–1.26]	1.53 [0.75–3.12]	1.45 [0.68–3.10]	1.25 [0.67–2.32]	1.36 [0.81–2.30]
Clinic B (vs. Clinic D)	0.66 [0.43–1.02]	1.08 [0.53–2.20]	1.86 [0.92–3.76]	1.36 [0.76–2.44]	1.26 [0.76–2.08]
Clinic C (vs. Clinic D)	0.87 [0.54–1.40]	0.98 [0.45–2.15]	1.79 [0.87–3.69]	0.79 [0.41–1.55]	0.98 [0.56–1.70]

228 cases were imputed for missing data on mother’s education**.** OR = odds ratio; CI = confidence interval; AOD = alcohol or drug use; PESQ = Personal Experience Screening Questionnaire; Analyses are adjusted for clustering within clinic; *p < .05; **p < .01; ***p < .001

Compared with the set of models that excluded the main effect of MHI-5 (not shown), including MHI-5 in the model increased the associations between AOD risk and AOD screening (ΔOR = 0.06) and advice to cut-down or stop for red flag (ΔOR = 0.43), but decreased the associations for referral (ΔOR = −0.75), brief counseling (ΔOR = -0.67), and screening plus any intervention (ΔOR = −0.56) for red flag. However, the pattern of significance for the AOD risk associations was generally similar across the model specifications.

In terms of demographic associations with screening or intervention, being older was associated with higher odds of being screened (OR = 1.25, *p* < .001) but lower odds of being counseled (OR = 0.88, *p* < .05). Black (OR = 0.42, p < .001), Hispanic (OR = 0.40, *p* < 0.001) and Multiracial or other non-white race/ethnicity (OR = 0.56, *p* < .01) adolescents had lower odds of being screened. We found no significant interactions between AOD risk and MHI-5 (not shown) for the set of models that added the interaction terms.

## Discussion

Adolescent AOD risk is a continuing concern. The PC setting provides an optimal opportunity for querying adolescents about risk behavior, including AOD use. This study contributes new information to the literature on adolescent AOD screening by examining both AOD use and mental health status together. Another contribution of this study is its examination of a large and diverse sample of underserved adolescents to understand the extent to which AOD risk and mental health influence the care that adolescents receive. Fully 85% of the sample is from diverse racial and ethnic backgrounds and unlike earlier studies includes adolescents from the full age range (12–18 year olds) incorporating those under age 16.

We found that consistent with previous research [19, 21, 22], overall rates of screening and intervention for adolescent AOD use were low. Specifically, less than two thirds reported being screened for use, and only a third of those at risk for AOD use (yellow or red flag on the PESQ) who were screened for AOD use reported receiving some type of intervention.

We also found that the odds of being screened or receiving brief intervention for AOD use increased for adolescents who were at highest risk. This suggests that medical providers in PC are identifying and addressing those who are more dependent on substances. We did not find, however, that having both higher risk of AOD use and poorer mental health increased the odds of receiving screening and intervention. Our models that included the MHI-5 intensified the associations between AOD risk for screening and advice outcome measures but attenuated the associations for referral, counseling, or screening plus an intervention. This suggests that medical providers are identifying risky AOD use *independent* of mental health problems and that poorer mental health may also be a trigger for more screening and advice about AOD use. Given the high rates of comorbidity between AOD use and mental health among adolescents, adolescents identified as high-risk for both problems may benefit from referral to integrated models of care.

We also observed significant associations between demographic characteristics and reported care. Older adolescents were more likely to report being screened, but less likely to report being counseled which could be explained by the higher rates of use among older adolescents. Adolescents from racial/ethnic minority groups were less likely to report being screened. This suggests potential disparities in care across race/ethnicity.

This study has several strengths. It is the first to examine the likelihood of adolescents’ receipt of care from a medical provider for AOD risk for varying levels of risk with a large and diverse population, as well as examining the additive effect of having poor mental health on reported care. Nevertheless, findings must be interpreted with a few caveats. The outcome measures of AOD screening and intervention are based on adolescent self-reports, which could be biased [36]. Further, we relied on a one year retrospective reporting period, which, given its length, could limit accuracy. For example, cognitive factors such as poor recall or comprehension [37] could compromise accuracy of responses and situational factors including social desirability [38], and perceived lack of confidentiality could also lead to underreporting of risky behavior. However, we designed clinic recruitment procedures for maximum confidentiality by securing private space to conduct assessments for study eligibility. Further, surveys were web-based self-reports that could be completed in private areas. We also included a fictitious drug in the survey and very few respondents reported taking it (only 10 of 1573 adolescents), which suggests that reporting was not necessarily biased [39] and estimates of AOD use in our sample match national survey norms [40]. Finally, this U.S. based study may not generalize to other areas in the U.S. or to other countries with different healthcare systems.

We also cannot determine whether the large number of adolescents who did not report receiving any form of intervention was due to a positive screening but no care or because youth were screened and deemed not to be at risk. Despite this uncertainty, we know that adolescents in the red flag group, who were current AOD users, reported not being screened for use e.g., less than 100% of those in need were screened. This suggests that PC is missing opportunities to identify adolescents at risk and intervene with them early in their lifespan to prevent future consequences of prolonged AOD use. Finally, because of the way in which the screening and intervention question was worded, we cannot separate out counseling for AOD use from counseling for mental health problems. Future research is needed to disentangle counseling for AOD use and from mental health counseling.

With continuing high prevalence of AOD use among adolescents, strategies for facilitating the opportunity to intervene and educate adolescents in primary care about AOD use may be warranted. These strategies should address some of the barriers to screening and intervention by providing organizational support and training [24] so that PC can more easily integrate screening into the adolescent’s appointment and ultimately prevent or delay AOD use into adulthood when it is less risky. Such efforts, however, should acknowledge that there is inconclusive evidence for screening’s effectiveness in reducing substance use among adolescents [41], and also recognize that PC providers may screen; however, competing time demands [42, 43] and/or limited training [23] may prevent them from being able to deliver effective interventions.

Although less accurate than validated interviewer-administered diagnostic instruments, single item screening questions (SISQs) are a promising approach to screening for risky health behaviors in PC settings because they are both practical and feasible [44–46]. SISQs are sufficiently brief for incorporation into busy practice settings, are less likely to be biased by social desirability, and also may facilitate conversations in a less stigmatized manner [47, 48]. Recently, a study validated the use of a self-administered SISQ for AOD use on a tablet for adult primary care patients [49]. Tablet administration decreases provider burden and may lessen stigma for patients compared with responding to provider questions directly. Such an approach could be broadly incorporated into primary care practice and may be adaptable for use with adolescents. Moreover, such a screening approach could decrease discomfort associated with discussing a sensitive topic and would also be time efficient.

However, screening alone is not enough. It must be paired with approaches for delivering brief interventions in the primary care setting. Consistent with Sterling et al. (2012) [24] implementing clinical practices and policies to address the barriers to intervention are needed. These include restructuring practice to allow for extra time needed for encounters with at-risk adolescents, such as flexible scheduling, reimbursement for longer visits with at-risk patients, or advance visit self-screening. Other facilitators to screening and intervention may include additional training for medical providers to increase comfort with addressing AOD use (e.g., de-stigmatize) [50, 51], as well as addressing concerns regarding patient confidentiality and adverse consequences of documenting AOD use in the medical record. Intervening in as little as 15 min can lead to positive behavior change [52]. Additionally, future research should examine the relative effectiveness of the screening, brief intervention, and referral to treatment model [53, 54] for risky AOD use to facilitate provider competency for delivering such care for adolescents in PC settings. Finally, given the high rates of comorbidity between AOD use and mental health issues, strategies are needed to facilitate medical providers’ understanding of the importance of potential identifying the need for counseling of both AOD and mental health care for at risk youth.

## Conclusion

Risky AOD use by adolescents remains a concern, particularly among adolescents with poor mental health functioning. Although there are barriers to screening [23–26], many providers are aware of the important role of screening for both of these problems so that adolescents may be referred to needed services. While screening rates were low in this large and diverse sample of adolescents, those at highest risk were significantly more likely to be screened and receive intervention. Findings highlight the continued need to decrease barriers in PC settings for screening and intervention with adolescents at risk for AOD use. Older adolescents were more likely to be screened but not to receive intervention, and were actually less likely to receive counseling. Adolescents from minority groups were significantly less likely to be screened than white adolescents but race/ethnicity was not associated with receipt of any type of intervention.

These findings underscore the importance of identifying practical, effective, and cost-effective strategies for addressing AOD risk among adolescents in PC settings. Further, to ensure that adolescents who screen positive for AOD use receive some sort of intervention and further assessment of mental health status, practice-based strategies that support the extra time needed for at-risk youth through flexible scheduling and reimbursement for expanded encounter durations are needed. Finally, strategies for facilitating PC provider competency including training about how to screen in a sensitive manner that feels comfortable and addresses issues of stigma, perceived confidentiality, and potential adverse consequences of patient disclosure are needed.

## Abbreviations

AOD: Alcohol and Other Drug use
MHI-5: 5-Item Mental Health Inventory
NIAAA: National Institute on Alcohol Abuse and Alcoholism
OR: Odds Ratio
PESQ-PS: Personal Experience Screening Questionnaire-Problem Severity Scale
SAS 9.3: Statistical Analysis Software version 0.3
SISQ: Single Item Screening Questions
